# Positive 14-3-3 protein in cerebrospinal fluid followed by poppy-induced delayed post-hypoxic leukoencephalopathy: A case report

**DOI:** 10.1016/j.heliyon.2024.e37129

**Published:** 2024-08-30

**Authors:** Guangxun Shen, Hanrong Dong, Jingmin Zhao, Si Wu, Kwee-Yum Lee, Lumei Chi

**Affiliations:** a Department of Neurology, China-Japan Union Hospital of Jilin University, Changchun, China; b Department of Orthopaedic Surgery and Musculoskeletal Medicine, University of Otago, Christchurch, New Zealand

**Keywords:** Delayed post-hypoxic leukoencephalopathy, Magnetic resonance imaging, Choreoathetosis, Progressive cognitive decline, 14-3-3 protein

## Abstract

**Background:**

Delayed post-hypoxic leukoencephalopathy (DPHL) is characterized by a biphasic clinical course, with complete recovery from coma to a fully conscious state lasting one to four weeks (lucid interval), followed by abrupt neurological deterioration as an indirect consequence of hypoxic events like carbon monoxide poisoning and narcotic drug overdose. To our best knowledge, there are no documented cases in literature of choreoathetosis and dementia following poppy-induced DPHL with 14-3-3 protein in cerebrospinal fluid (CSF).

**Case presentation:**

We report the case of a 70-year-old female who underwent cardiopulmonary resuscitation (CPR) due to overdose of homemade refined opium poppy paste two weeks prior to presentation. She presented a progressive cognitive decline, along with the development of apraxia and choreic movement affecting her tongue and bilateral upper and lower extremities. During the symptomatic phase, brain magnetic resonance imaging (MRI) showed bilateral symmetrical hyperintense signals mostly in central frontal, temporal, and parieto-occipital lobes in the diffusion weighted imaging (DWI) and fluid-attenuated inversion recovery (FLAIR) sequences which are the characteristic findings of DPHL. CSF routine analysis, as well as toxicology screening, autoimmune and paraneoplastic encephalitis panels were negative, but the presence of 14-3-3 protein in the CSF was detected. With steroid therapy, hyperbaric oxygen therapy and symptomatic treatment, she experienced gradual improvement in cognition, motivation, and psychomotor function.

**Conclusion:**

DPHL represents a distinct form of encephalopathy characterized by unique clinical course and imaging features. It is the first report of DPHL with positive 14-3-3 protein in CSF. The potential of 14-3-3 protein as a biomarker for diagnosing DPHL and its ability to predict disease severity and prognosis warrants further research.

## 1 Introduction

Delayed post-hypoxic leukoencephalopathy (DPHL) is a condition characterized by a biphasic clinical course. Initially, patients experience a complete recovery from the acute hypoxic episode, returning to a fully conscious state lasting one to four weeks, known as the lucid interval. This is followed by an abrupt neurological deterioration, which occurs as an indirect consequence of hypoxic events, such as carbon monoxide (CO) poisoning or respiratory failure in drug overdoses [1,2]. Typical symptoms of DPHL include onset of acute neuropsychiatric symptoms like confusion, disorientation, dyskinesia such as parkinsonism and akinetic mutism [3]. Herein, we present a rare manifestation of DPHL where the patient demonstrated choreoathetosis and dementia following poppy-induced DPHL. Notably, we report the first-ever detection of 14-3-3 protein in cerebrospinal fluid (CSF) of the patient diagnosed with DPHL.

## 2 Case presentation

**Fig. 1:**
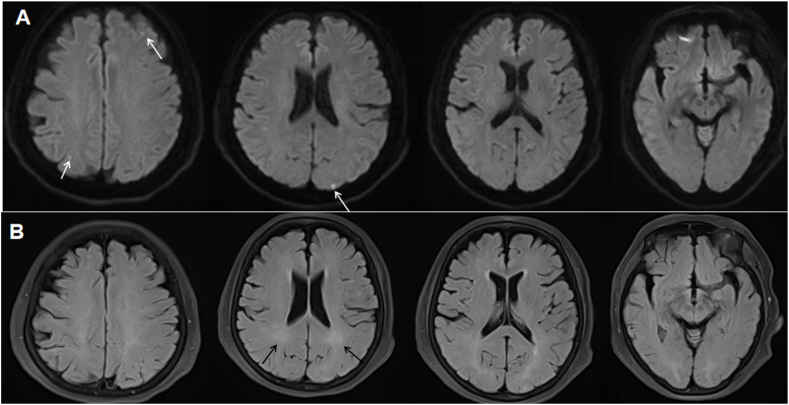
MRI findings at coma status following a hypoxic event induced by poppy overdose. DWI showed acute multifocal punctate infarcts (white arrows) (A) and FLAIR sequence demonstrated mild white matter demyelination (black arrows) (B). MRI = magnetic resonance imaging; DWI = diffusion weighted imaging; FLAIR = fluid-attenuated inversion recovery.

**Fig. 2:**
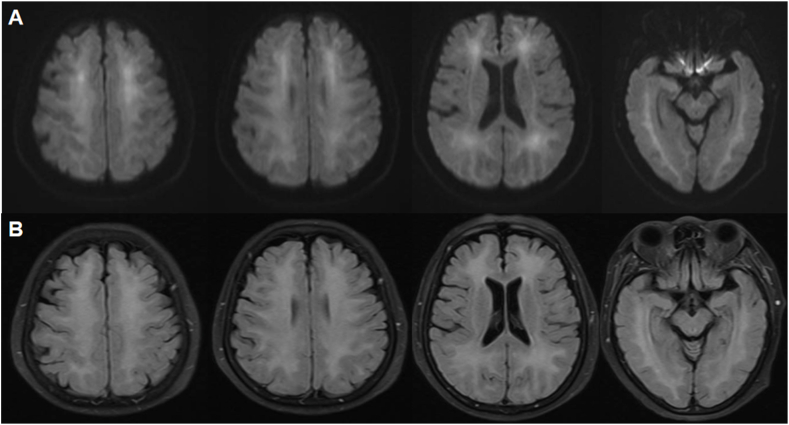
MRI findings at the presence of choreic movements 15 days after hypoxic events. DWI(A) and FLAIR (B) sequences revealed symmetrical diffuse hyperintense signals predominantly in central white matter of bilateral frontal, temporal, parieto-occipital lobes. MRI = magnetic resonance imaging; DWI = diffusion weighted imaging; FLAIR = fluid-attenuated inversion recovery.

**Fig. 3:**
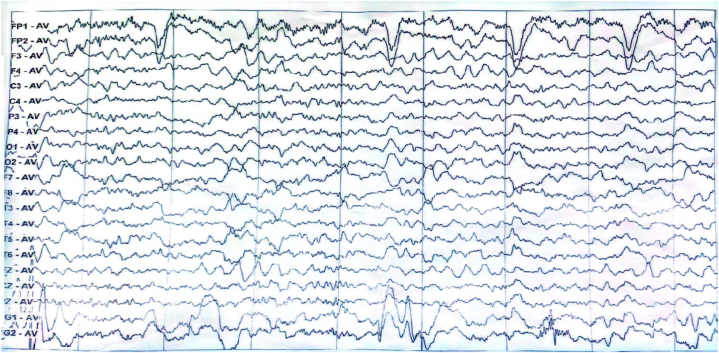
EEG findings of poppy-induced DPHL. EEG showed a mixed slow wave activity of low and middle amplitude, along with paroxysmal irregular theta rhythm in the bilateral frontal region. EEG = Electroencephalography; DPHL = delayed post-hypoxic leukoencephalopathy.

A 70-year-old female without notable medical history was admitted to cardiology department of our hospital in a comatose state. The patient's condition was attributed to a cardiac arrest probably induced by an overdose of homemade refined opium poppy paste, which had been prepared 20 years ago. She was diagnosed with acute non-ST-segment elevation myocardial infarction with significantly elevated T-troponin levels. Brain magnetic resonance imaging (MRI) showed facute multifocal punctate infarcts in the diffusion weighted imaging (DWI) (white arrows, Fig. 1A) and mild white matter demyelination in the T2 and fluid-attenuated inversion recovery (FLAIR) sequence (black arrows, Fig. 1B). Under antiplatelet therapy, the patient experienced a gradual resolution of symptoms and achieved full recovery within one week. On the eighth day following her discharge from hospital, she began to experience a progressive choreic movements, cognitive decline, apraxia and impaired comprehension. The deterioration progressed to the point where she became unable to carry out routine daily activities. Clinical examination revealed generalized choreic movement affecting her tongue and bilateral upper and lower extremities (Supplementary Video 1.). Plantar reflexes were flexor bilaterally and no focal deficit in strength was observed. The patient's hemogram, blood sugar level, liver and renal function test, HIV and VDRL results were negative. Toxicology screening, CSF routine analysis, autoimmune and paraneoplastic encephalitis panel were negative. However, the presence of 14-3-3 protein was detected by Western blot in the CSF(See supplementary materials for details). The MRI showed bilateral symmetrical hyperintense signals mostly in central frontal, temporal, and parieto-occipital lobes in the DWI (Fig. 2A) and FLAIR (Fig. 2B) which are the characteristic findings of DPHL. The internal capsule, periventricular region, cerebellum, and brainstem appeared unaffected. Electroencephalography (EEG) showed a mixed slow wave activity of low and middle amplitude, along with paroxysmal irregular theta rhythm in the bilateral frontal region (Fig. 3). Despite receiving thirteen days of steroid therapy, hyperbaric oxygen therapy and tiapride 100mg three times a day, in addition to haloperidol 4mg three times a day, the patient did not show much improvement. However, a notable recovery occurred when deutetrabenazine 6mg once a day was given, resulting in remarkable improvement in her choreic movement. Over the course of 30 days following the onset, her cognitive ability gradually recovered. At the six months follow-up, she was able to carry out normal activities.

Supplementary video related to this article can be found at https://doi.org/10.1016/j.heliyon.2024.e37129

The following is/are the supplementary data related to this article:

Video 1 2 Video 1

## 3 Discussion

DPHL is an underrecognized entity because there are no widely accepted formal criteria for its diagnosis. Only with appropriate clinical history and supplemental testing excluding alternative causes of acute altered mental status can the diagnosis be confirmed [4]. In our case, rapidly progressive cognitive dysfunction, extrapyramidal manifestations, bilateral cortical hyperintensities in DWI and the presence of 14-3-3 protein in the CSF were consistent with the diagnostic criteria for sCJD. However, the absence of the typical DWI/MRI cortical ribboning and paroxysmal irregular theta rhythm in the bilateral frontal region of EEG suggested against a sCJD diagnosis. The diagnosis of DPHL is confirmed in our case due to the following reasons: 1) the clinical course aligns with typical pattern of DPHL, characterized by an initial event of a lucid interval and subsequent presentation of progressive cognitive decline, apraxia, and choreoathetosis; 2) The patient had characteristic imaging findings of DPHL on brain MRI, revealing bilateral diffuse white matter demyelination; 3) Supplemental testing provided have ruled out alternative diagnoses. Similarly, Kawasaki J et al. reported a 45-year-old woman presented a series of neurological dysfunctions which were finally diagnosed as DPHL by characteristic MRI as the result of cerebral hypoxia and hypoperfusion due to the dyspnea caused by severe COVID-19,who reached a full recovery with persistent systemic support [5]. Jingami N et al. endorsed hyperbaric oxygen therapy for a 47-year-old male with severe disturbances in consciousness because of opioid intoxication, which improved cerebral blood flow and oxygenation [6]. Pfaff JAR et al. presented a rare case where the patient presented clinical symptoms suggestive of DPHL following successful revascularization therapy for large vessel occlusions, whose radiological signs of DPHL were subtle and unilateral, strictly confined and localized unilaterally to the left anterior circulation, offering a clear example of DPHL resulting from oxygen deprivation [7].

**Fig. 4:**
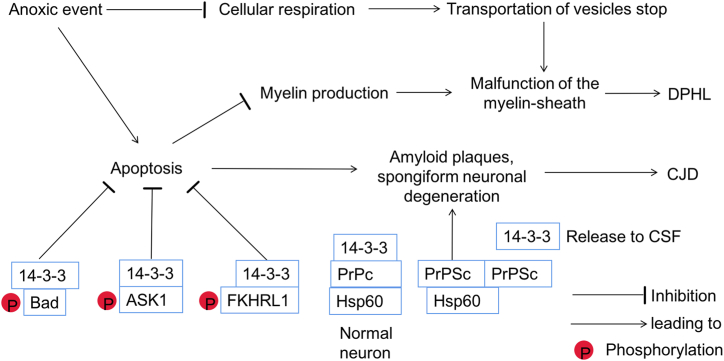
Pathway of DPHL and possible common pathway between sCJD and DPHL. Anoxic event inhibits normal functioning of cellular respiration, thus the transportation of vesicles stops, leading to malfunction of the myelin-sheath. Anoxic event promotes the apoptosis of oligodendrocytes, which are responsible for myelin production, causing malfunction of the myelin-sheath.14-3-3 proteins inhibit apoptosis through three pathways, which could be a possible pathway for spongiform neuronal degeneration in CJD. The phosphorylated Bad by AKT combines with 14-3-3 proteins, stopping BAD from entering the mitochondra, then suppresing apoptosis. By forming a complex with 14-3-3 proteins after the phosphorylation, ASK1 restrains the activity of ASK1 kinase, resulting in inhibition of apoptosis. After phosphorylation at site Thr24, FKHRL1 can be reognized by the 14-3-3 proteins, thereby inhibiting FKHRL1-induced apoptosis. Under physiological conditions, the ternary complex of 14-3-3 proteins, heat shock protein 60 (Hsp60) and PrPc might prevent PrPc from the autocatalytic conformational change. When infected by pathogenetic prion, 14-3-3 protein and PrPc is replaced by PrPSc aggregates, which is facilitated by Hsp60. As a result, the 14-3-3 proteins releasing from degenerating neurons could be detected in the CSF, leading to the characteristic amyloid plaques, spongiform neuronal degeneration.

The precise underlying pathology of DPHL remains elusive, but several theories have been proposed [8]. Firstly, the disruption of adenosine triphosphate (ATP)-based cellular respiration could play a role in the development of specific pathological changes in the white matter. Factors such as opioid overdose, CO poisoning, cyanide poisoning and hypoxic environments pose significant threats to the normal functioning of cellular respiration [9]. Under such circumstances, the transportation of vesicles containing nutrients, neurotransmitters, and vital substances from axons to dendrites may become restricted. As the substances stored in the distal ends of dendrites are depleted, the accumulation of toxin leads to malfunction of the myelin-sheath, which accounts for the delayed onset of symptoms in DPHL (Fig. 4). Subsequently, when the supply of oxygen is sufficient, the entire process is normalized, resulting in a likelihood of full recovery and spontaneous remission of DPHL. Supporting this theory is the strong association between the cycle of myelin-related proteins and emergence of clinical symptoms. Apoptosis, a selective and programmed cell death, has been proposed as another underlying mechanism in DPHL. In the context of DPHL, apoptosis specifically affects oligodendrocytes, which are responsible for myelin production, and is triggered by external threats or internal disruptions within the cellular environment [10]. Compared to other demyelination disease that often result in irreversible and uncontrollable damage, DPHL exhibits an elaborate process of white matter change, which offers a more favorable prognosis (Fig. 4).

Notably, this is the first report of 14-3-3 protein being detected in the CSF of patient with DPHL. Traditionally, 14-3-3 protein in CSF has been strongly associated with Creutzfeldt-Jakob Disease (CJD), exhibiting high sensitivity and specificity for this condition (sensitivity 92 % and a specificity of 80 %) [11]. 14-3-3 proteins inhibit apoptosis through three pathways, which could be a possible pathway for spongiform neuronal degeneration in CJD. The phosphorylated Bad by AKT combines with 14-3-3 proteins, stopping BAD from entering the mitochondra, then suppresing apoptosis. By forming a complex with 14-3-3 proteins after the phosphorylation, ASK1 restrains the activity of ASK1 kinase, resulting in inhibition of apoptosis. After phosphorylation at site Thr24, FKHRL1 can be reognized by the 14-3-3 proteins, thereby inhibiting FKHRL1-induced apoptosis [12]. Under physiological conditions, the ternary complex of 14-3-3 proteins, heat shock protein 60 (Hsp60) and PrPc might prevent PrPc from the autocatalytic conformational change. When infected by pathogenetic prion, 14-3-3 protein and PrPc is replaced by PrPSc aggregates, which is facilitated by Hsp60. As a result, the 14-3-3 proteins releasing from degenerating neurons could be detected in the CSF, leading to the characteristic amyloid plaques, spongiform neuronal degeneration [13] (Fig. 4).

However, there is an expanding range of neurological conditions unrelated to prion diseases, including anoxic, metabolic or drug-induced encephalopathy [14]. Lee D et al. presumed that 14-3-3 protein may protect oligodendrocyte against autoimmune demyelination through immediating apoptosis [15]. In ischemic cortical neurons, 14-3-3 protein reduced oxygen glucose deprivation-induced cell death by binding to a phosphorylated BAD, preventing its entry into the mitochondria and consequently inhibiting the initiation of apoptosis [16]. Furthermore, in COVID-19 associated encephalopathy, levels of 14-3-3 protein has been found to be closely related with the neurological status of patients throughout an 18-months of follow-up period. This finding suggests that elevated levels of 14-3-3 protein may be indicative of significant brain damage in these conditions [17]. Beretta S et al. reported a peculiar type of immune-mediated encephalitis related to COVID-19 infection which closely resembles acute-onset sCJD with a positive 14-3-3 protein detected in the CSF [18]. Therefore, the protective mechanism of 14-3-3 protein seems to involve the pathways linked to both apoptosis and immune responses. Based on our case, it is reasonable to presume that poppy-induced DPHL results in significant brain damage mediating by apoptosis, causing the release of 14-3-3 protein.

There has not yet effective treatment for DPHL. Immunotherapy (glucocortieoid pulse therapy and plasma exchange) has yielded unfavorable outcomes in other countries [19], while hyperbaric oxygen (HBO) may improve the prognosis of DPHL patients by enhancing myelin regeneration, even if the cause is not CO poisoning [20]. Combined with a N-butylphthalide (NBP) and dexamethasone (DXM), HBO could improve the cognitive and motor functions of DPHL patients caused by carbon monoxide poisoning [21]. Cree BAC et al. presented a case of severe DPHL who regained his ability to work in the use of clemastine on the basis that promoting the differentiation of oligodendrocyte precursors through inhibiting muscarinic pathway could preventing progressive neurological injury [22]. Therefore, more reseraches are warranted to explore the best treatment for DPHL.

## 4 Conclusion

In conclusion, DPHL represents a distinct form of encephalopathy characterized by unique clinical course and imaging features. The presence of elevated 14-3-3 protein in CSF may serve as a potential indicator of DPHL. However, further research is needed to determine whether 14-3-3 protein could be established as a reliable biomarker for diagnosing DPHL.

## Ethics statement

Written informed consent for the publication of this case report, including accompanying images and video, was obtained from the patient's daughter.

## Data availability statement

All data supporting the findings of this study are available within the paper and its Supplementary Information.

## CRediT authorship contribution statement

**Guangxun Shen:** Writing – review & editing, Writing – original draft, Conceptualization. **Hanrong Dong:** Writing – review & editing, Writing – original draft, Conceptualization. **Jingmin Zhao:** Writing – review & editing, Data curation. **Si Wu:** Writing – review & editing, Data curation, Conceptualization. **Kwee-Yum Lee:** Writing – review & editing, Data curation. **Lumei Chi:** Writing – review & editing, Writing – original draft, Conceptualization.

## Declaration of competing interest

The authors declare that they have no known competing financial interests or personal relationships that could have appeared to influence the work reported in this paper.
