# Supplementary material for: Positive 14-3-3 protein in cerebrospinal fluid followed by poppy-induced delayed post-hypoxic leukoencephalopathy: A case report
Source: Heliyon. 2024 Aug 30;10(17):e37129. doi: 10.1016/j.heliyon.2024.e37129 (PMC11407924; doi:10.1016/j.heliyon.2024.e37129)
Supplement: Multimedia component 1 [file mmc1.doc]

**Supplementary Video Legend:**

Video 1.: Clinical examination revealed generalized choreic movement affecting her tongue and bilateral upper and lower extremities.

**Supplementary Materials:**

14-3-3 protein immunoassay was performed by Western blot analysis. Briefly, 75 microliters of CSF samples were denatured in a solution containing Tris 0.05 M (pH 7.4), SDS 10%, DTT 1%, and glycerol, followed by heating at 98°C for 5 mins. The proteins were then separated using an ExcelGel® SDS Homogeneous 15 for SDS-PAGE (GE Healthcare®, Little Chalfont, Buckinghamshire, UK), transferred onto a polyvinylidene fluoride (PVDF) membrane, and blocked overnight with 1% nonfat-dried milk in Tris Buffer Saline (TBS). The membranes were incubated for 2 hrs with the primary anti-14.3.3 antibody at a 1:400 dilution (β K19: sc-629, Santa Cruz Biotechnology®, Dallas, TX, USA). Following incubation, the membranes were washed three times with TBS containing 0.1% nonfat dried milk. They were then saturated for 1 hr with an alkaline phosphatase conjugated anti-rabbit second antibody at a 1:1000 dilution (sc-2057, Santa Cruz Biotechnology®, Dallas, TX, USA) and subsequently washed three more times with TBS containing 0.1% nonfat dried milk, followed by a final wash with TBS. The colorimetric detection was then carried out by incubating the membrane with 5-bromo-4-chloro-3-indolyl-phosphate/nitro blue tetrazolium (Thermo Scientific®, Waltham, MA, USA) for 5 mins. The 14-3-3 protein band in the CSF samples was optically assessed and compared to known specimen to determine whether the sample was classified as positive or negative. Western Blot analysis can provide qualitative and semi-quantitative information about the protein, allowing us to distinguish between different 14-3-3 protein isomers. Specifically, we detected 14-3-3ζ, which have a molecular weight of 30kDa, confirmed by specific bands through antibody-mediated reactions and it was verified in the repeat the experiment.
